# Supplementary material for: Soil fungal networks maintain local dominance of ectomycorrhizal trees
Source: Nat Commun. 2020 May 26;11:2636. doi: 10.1038/s41467-020-16507-y (PMC7250933; doi:10.1038/s41467-020-16507-y)
Supplement: Supplementary file 1 — Supplementary Information [file 41467_2020_16507_MOESM1_ESM.pdf]

Supplementary Information for:

Soil fungal networks maintain local dominance of ectomycorrhizal trees

Liang et al.

Supplementary Tables

Supplementary Table 1: Results of the generalized linear mixed-effects model testing for the effect of different mesh sizes of in-growth cores on seedling survival of ECM and AM tree species.

Supplementary Table 2: Results of the linear mixed-effects model testing for the effect of different mesh sizes of in-growth cores on seedling total biomass of ECM and AM tree species.

Supplementary Table 3: Results of the linear mixed-effects model testing for the effect of different mesh sizes of in-growth cores on root mycorrhizal colonization rates of ECM and AM tree seedlings.

Supplementary Table 4: The list of focal tree species for the in-growth core experiments.

Supplementary Figures

Supplementary Figure 1: Comparisons of soil moisture content and soil temperature between 35 μm and 0.5 μm mesh cores for ECM and AM tree species.

Supplementary Figure 2: A comparison of the relative effect of conspecific and heterospecific sites on seedling growth between 35 μm or 0.5 μm mesh cores for ECM and AM tree species.

Supplementary Table 1. Results of the generalized linear mixed-effects model testing for the effect of different mesh sizes of in-growth cores on seedling survival of ECM and AM tree species.

Fixed effects	Estimate	SE	*z*	*P*
**Intercept**	**1.735**	**0.434**	**3.998**	**< 0.001**
Mesh size (35 µm)	-0.418	0.264	-1.582	0.114
Mycorrhizal type (ECM)	-0.636	0.595	-1.070	0.285
**Mesh size : Mycorrhizal type**	**1.302**	**0.380**	**3.430**	**< 0.001**

Bold values indicate significance at $P < 0.05$.

Supplementary Table 2. Results of the linear mixed-effects model testing for the effect of different mesh sizes of in-growth cores on seedling total biomass of ECM and AM tree species.

Fixed effects	Estimate	SE	*t*	*P*
Intercept	0.668	0.299	2.24	0.075
**Mesh size (35 µm)**	**0.125**	**0.039**	**3.18**	**0.002**
Site mycorrhizal type (ECM)	0.223	0.141	1.58	0.180
Seedling mycorrhizal type (ECM)	-0.210	0.400	-0.53	0.627
**Site mycorrhizal type : Seedling mycorrhizal type**	**-0.156**	**0.074**	**-2.12**	**0.035**

Bold values indicate significance at $P < 0.05$.

Supplementary Table 3. Results of the linear mixed-effects model testing for the effect of different mesh sizes of in-growth cores on root mycorrhizal colonization rates of ECM and AM tree seedlings. Seedlings were planted at either their own sites (home) or the five heterospecific sites (away) in the seedling growth experiment.

Fixed effects	Estimate	SE	*t*	*P*
**Intercept**	**0.472**	**0.113**	**4.17**	**< 0.001**
**Mesh size (35 µm)**	**0.148**	**0.062**	**2.39**	**0.017**
**Site type (Away site)**	**-0.302**	**0.072**	**-4.22**	**< 0.001**
Site mycorrhizal type (ECM)	0.053	0.102	0.53	0.607
Seedling mycorrhizal type (ECM)	-0.040	0.085	-0.47	0.652
**Mesh size : Site type**	**-0.265**	**0.076**	**-3.50**	**< 0.001**
**Site mycorrhizal type : Seedling mycorrhizal type**	**0.207**	**0.073**	**2.84**	**0.006**

Bold values indicate significance at $P < 0.05$.

Supplementary Table 4. The list of focal tree species for the in-growth core experiments.

Focal species	Family	Mycorrhizal type	Species code	Experiment in which used
Castanopsis faberi	Fagaceae	ECM	Cfab	Survival, Growth
Castanopsis fissa	Fagaceae	ECM	Cfis	Survival, Growth
Cyclobalanopsis hui	Fagaceae	ECM	Chui	Survival
Lithocarpus haipinii	Fagaceae	ECM	Lhai	Survival
Engelhardtia fenzelii	Juglandaceae	ECM	Efen	Growth
Schima superba	Theaceae	AM	Ssup	Survival, Growth
Cryptocarya concinna	Lauraceae	AM	Ccon	Survival, Growth
Canarium album	Burseraceae	AM	Calb	Survival
Ormosia glaberrima	Fabaceae	AM	Ogla	Survival
Artocarpus styracifolius	Moraceae	AM	Asty	Growth

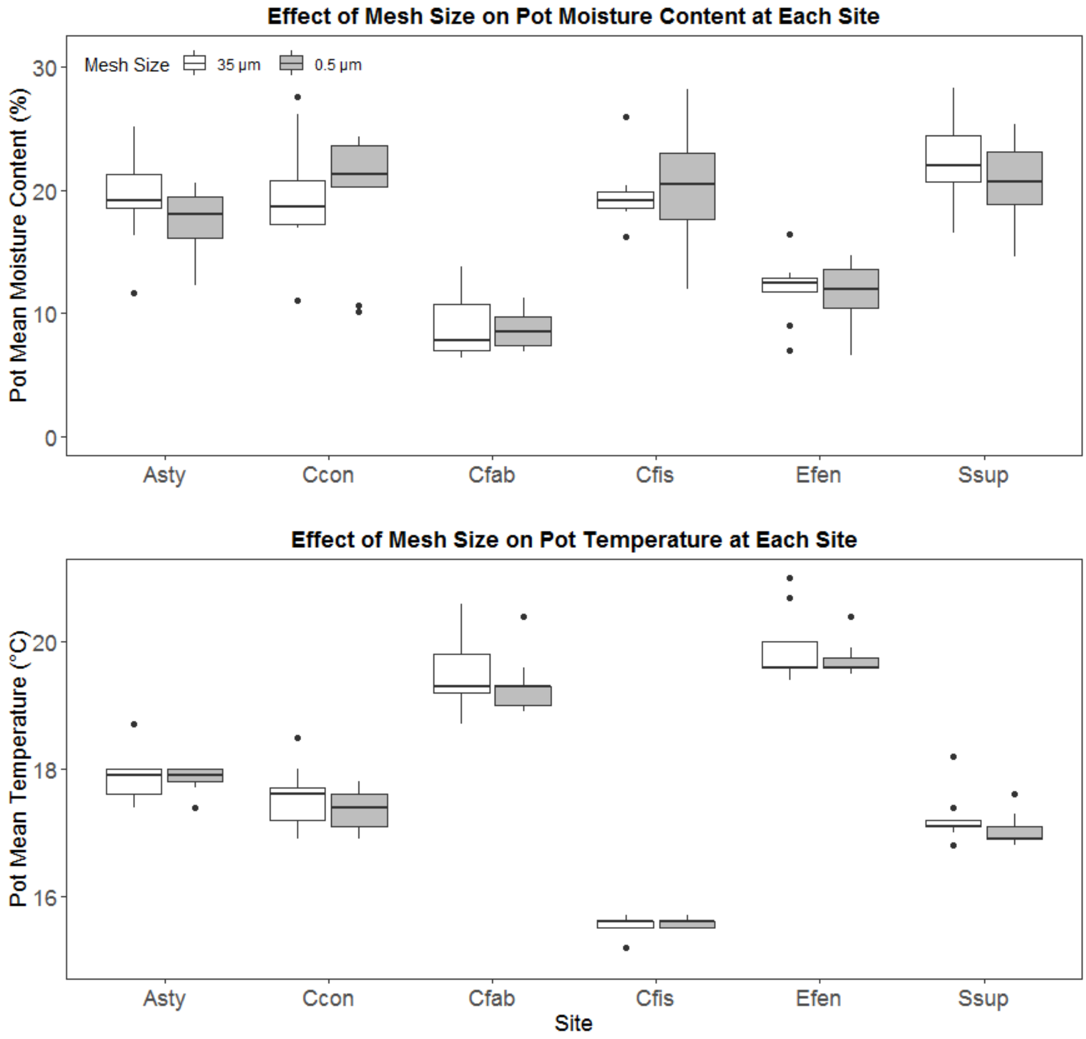

Supplementary Fig. 1. Comparisons of soil moisture content and soil temperature between 35 µm and 0.5 µm mesh cores for ECM and AM tree species. Soil moisture content and temperature using the HydraProbe Sensors (Stevens Water Monitoring Systems Inc., Portland, USA) for each in-growth core. The species 4-letter codes are as in Supplementary Table 4. Boxplots represent the variation across cores within mesh treatment and site, with the black line as the median, the boxes representing the quartiles, the whiskers 1.5 times the interquartile range and the dots the outlier points (n = 6 independent in-growth cores). Source data are provided in a Source Data file.

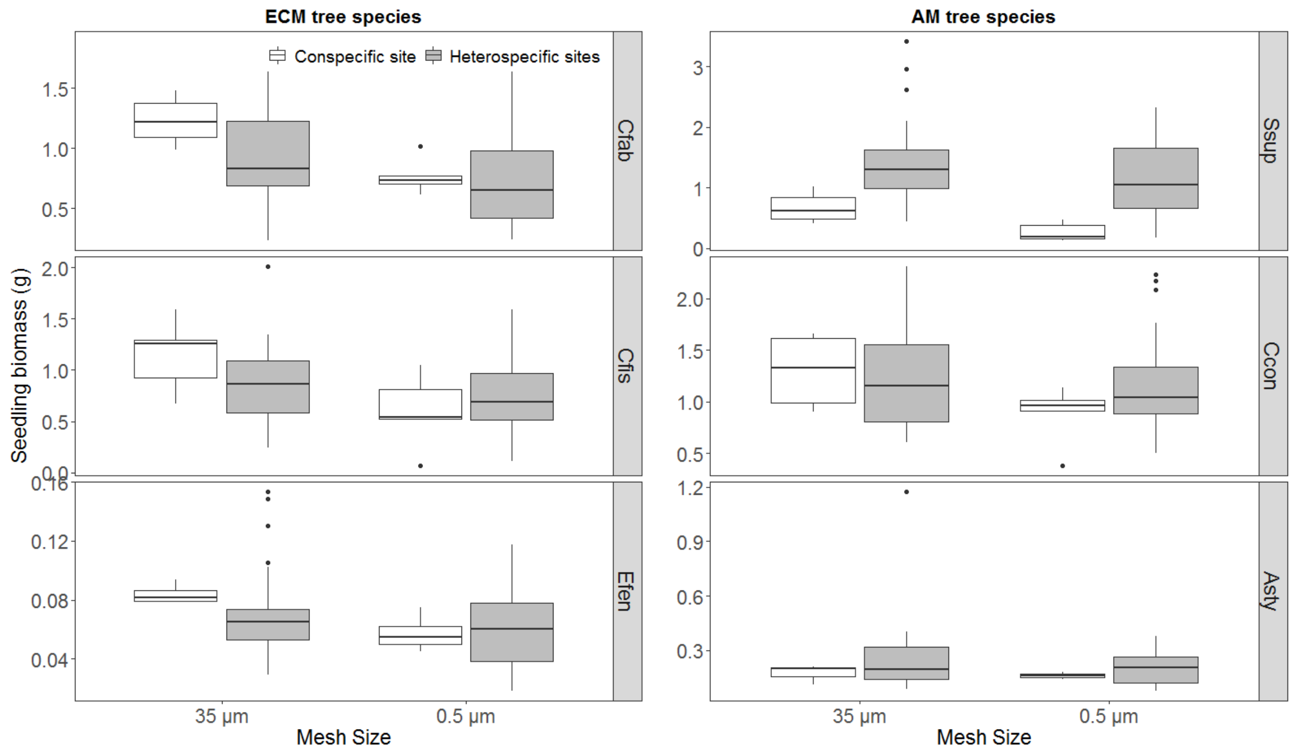

Supplementary Fig. 2. A comparison of the relative effect of conspecific and heterospecific sites on seedling growth between 35 µm or 0.5 µm mesh cores for ECM and AM tree species. Seedlings were planted at either their own sites (white) or the five heterospecific sites (grey). Boxplots represent the variation across cores within mesh treatment and site, with the black line as the median, the boxes representing the quartiles, the whiskers 1.5 times the interquartile range and the dots the outlier points (n = 6 biologically independent seedlings for conspecific sites and n = 30 biologically independent seedlings for heterospecific sites). Source data are provided in a Source Data file.
