# Supplementary material for: Soil fungal networks maintain local dominance of ectomycorrhizal trees
Source: Nat Commun. 2020 May 26;11:2636. doi: 10.1038/s41467-020-16507-y (PMC7250933; doi:10.1038/s41467-020-16507-y)
Supplement: Supplementary file 3 — Reporting Summary [file 41467_2020_16507_MOESM3_ESM.pdf]

nature research

Corresponding author(s): Xubing Liu
Last updated by author(s): Apr 24, 2020

Reporting Summary

Nature Research wishes to improve the reproducibility of the work that we publish. This form provides structure for consistency and transparency in reporting. For further information on Nature Research policies, see Authors & Referees and the Editorial Policy Checklist .

Statistics

For all statistical analyses, confirm that the following items are present in the figure legend, table legend, main text, or Methods section.

n/a	Confirmed	
☐	☒	The exact sample size (*n*) for each experimental group/condition, given as a discrete number and unit of measurement
☐	☒	A statement on whether measurements were taken from distinct samples or whether the same sample was measured repeatedly
☐	☒	The statistical test(s) used AND whether they are one- or two-sided<br>*Only common tests should be described solely by name; describe more complex techniques in the Methods section.*
☐	☒	A description of all covariates tested
☐	☒	A description of any assumptions or corrections, such as tests of normality and adjustment for multiple comparisons
☐	☒	A full description of the statistical parameters including central tendency (e.g. means) or other basic estimates (e.g. regression coefficient) AND variation (e.g. standard deviation) or associated estimates of uncertainty (e.g. confidence intervals)
☐	☒	For null hypothesis testing, the test statistic (e.g. *F*, *t*, *r*) with confidence intervals, effect sizes, degrees of freedom and *P* value noted<br>*Give P values as exact values whenever suitable.*
☒	☐	For Bayesian analysis, information on the choice of priors and Markov chain Monte Carlo settings
☒	☐	For hierarchical and complex designs, identification of the appropriate level for tests and full reporting of outcomes
☒	☐	Estimates of effect sizes (e.g. Cohen's *d*, Pearson's *r*), indicating how they were calculated

Our web collection on statistics for biologists contains articles on many of the points above.

Software and code

Policy information about availability of computer code

Data collection	No software or code was used for data collection.
Data analysis	Raw sequence data was processed using MOTHUR (v 1.29), UCHIME (v 4.1), UPARSE (v 7.0.1001), QIIME (v 1.9.1) and FunGuild algorithm (http://www.funguild.org/).<br>All the data analyses were conducted using R (v 3.5.1) <http://www.R-project.org> and the R packages “lme4” (v 1.1-21), "ggplot2" (v 3.5.2).<br>Code Availability: The R code used in this study are available at https://github.com/liuxubing/common-mycorrhizal-networks.

For manuscripts utilizing custom algorithms or software that are central to the research but not yet described in published literature, software must be made available to editors/reviewers. We strongly encourage code deposition in a community repository (e.g. GitHub). See the Nature Research guidelines for submitting code & software for further information.

Data

Policy information about availability of data

All manuscripts must include a data availability statement. This statement should provide the following information, where applicable:
- Accession codes, unique identifiers, or web links for publicly available datasets
- A list of figures that have associated raw data
- A description of any restrictions on data availability

Shade-house experimental data are available in the NERC Environmental Information Data Centre at https://doi.org/10.5285/f1d17e61-bb6c-47a9-a648-062c63ea7f16. Sequence data have been deposited in the NCBI Sequence Read Archive (SRA) under Project ID PRJNA627300. Field census data are available upon reasonable request from the ForestGEO data portal at http://ctfs.si.edu/datarequest/.

Field-specific reporting

Please select the one below that is the best fit for your research. If you are not sure, read the appropriate sections before making your selection.

☐ Life sciences ☐ Behavioural & social sciences ☒ Ecological, evolutionary & environmental sciences

For a reference copy of the document with all sections, see nature.com/documents/nr-reporting-summary-flat.pdf

Ecological, evolutionary & environmental sciences study design

All studies must disclose on these points even when the disclosure is negative.

Study description

In this study, we tested the prediction that differential access to root-associated fungal networks regulates PSFs, and quantify how access to root-associated fungal connections affects growth, survival and pathogen loads of seedlings in patches of subtropical forest dominated by adult trees that associate with either ECM or AM fungi.

We conducted a seedling monitoring over ten years in the field, and two hyphal exclusion experiments with in-growth cores. For the seedling survival experiment, seedlings of eight species were transplanted to the sites dominated by their conspecific adults, with 6 replicate cores for each mesh size and 8 seedlings per core. For the seedling growth experiment, seedlings of six species were transplanted to each site dominated by a single focal species using a full reciprocal design. Treatments consisted of: (1) six focal species comprising three ECM and three AM species; (2) six corresponding sites dominated by one of the focal species; (3) two mesh sizes of cores (35 µm vs. 0.5 µm).

Research sample

Field census - We established 1,200 1-m2 seedling quadrats which were regularly distributed within six, 1-ha permanent plots in a subtropical forest at the Heishiding Nature Reserve of south China, and monitored survival of all woody plant seedlings within these quadrats every spring from 2008 to 2017. These long-term field censuses on naturally emerged tree seedlings allow us to investigate the strength of density-dependent effects on seedling recruitment. We targeted all 130 species in these quadrats, which represent the whole subtropical evergreen forests and could be used to make comparisons between ECM and AM species.

Seedling experiments - We selected ten common tree species in the study area as focal species, including five ECM species: Castanopsis fabri (Fagaceae), Castanopsis fissa (Fagaceae), Cyclobalanopsis hui (Fagaceae), Lithocarpus haipinii (Fagaceae), and Engelhardia fenzelii (Juglandaceae), and five AM species: Schima superba (Theaceae), Cryptocarya concinna (Lauraceae), Canarium album (Burseraceae), Ormosia glaberrima (Fabaceae), and Artocarpus styracifolius (Moraceae). These focal species were selected based on their mycorrhizal type, local abundance and seed availability at the time of collection. Both ECM and AM species were meant to represent each mycorrhizal type of subtropical trees respectively.

Sampling strategy

We recorded 17,824 individuals belonging to 130 species, 82 genera and 48 families over the ten years. The large number of monitored individuals, and the unprecedented temporal and spatial scales, could fully reveal long-term seedling dynamics in the subtropical forest, and also provide sufficient sample sizes to make comparisons on seedling dynamics between ECM and AM tree species.

The targeted species for seedling experiments were selected based on their abundance and seed availability at the time of collection, and the phylogenetic imbalance between ECM and AM species objectively represented the community composition at the field site, i.e. AM are widespread among tree species and ECM are restricted to fewer tree taxa, i.e. AM are widespread among tree species and ECM are restricted to fewer tree taxa. We transplanted and sampled 432 seedlings in total (n = 6 sites × 6 species × 2 mesh sizes × 6 seedlings per core)in the seedling growth experiment. A total of 768 seedlings (n = 8 species × 2 mesh treatments × 6 core replicates × 8 seedlings per core) were transplanted and sampled in the survival experiment. In total 1200 seedlings belonging to 10 focal species were analysed, which makes the results we observed in filed manipulative experiments convincing, given that most of previous common mycorrhizal network studies focused on single "one host - one fungi" system.

Data collection

Field census - We tagged all tree individuals with a diameter at breast height (DBH) ≥ 1 cm in the 1-ha plots. We identified their species, mapped their locations, and measured their DBH. In the seedling quadrats, we surveyed all seedlings of woody plants (from new emergents to DBH < 1 cm) every spring from 2008 to 2017. At each census, we tagged new seedlings, determined their species, and measured their heights. (Drs. Minxia Liang and Xubing Liu, with help from many lab members and research assistances as acknowledged in the main text)

Seedling experiments - At the end of both experiments, we removed each core from the soil and then carefully removed the seedlings. Each seedling was washed to remove any attached soil and separated into shoot and root, and their dry weights measured after oven-drying at 60 °C for 72 hours. At the harvest, we collected fresh root and soil samples from each core for laboratory analysis. We randomly collected 10 fine root fragments of 1 cm length from each seedling, and stored them in centrifuge tubes with a piece of wet filter paper in the bottom, kept at 4 °C and transferred to a laboratory within two days for analysis of mycorrhizal colonization. Mycorrhizal colonization of roots among focal species was quantified using the grid-line intersection method. AM colonization was defined as typically aseptate hyphae, forming arbuscules and vesicles, and ECM colonization was quantified by counting the percentage of colonized root tips covered with fungal mantle and Hartig net. (Dr. Minxia Liang, Mr. Miao Fang and Dr. Xubing Liu)

Timing and spatial scale

Field census - Seedling censuses were repeated annually every spring from 2008 to 2017, at the beginning of wet season with most seedlings germinated. All seedling quadarts were 1 × 1 m and regularly spaced within six 1-ha (100 × 100 m) permanent plots at the field site. Each census was conducted continuously for two to three weeks depending on weather.

Seedling experiments - The seedling growth experiment was conducted between March 2017 and January 2018 with six tree species, and the survival experiment was conducted from March to August 2018 with eight species. These time periods were chose mainly based on the seedling availability. We used newly germinated seedlings for the survival experiment to investigate the early-stage survival after seedling germination, but seedlings were cultured for one year before being used for the growth experiment, to maximize competition among seedling individuals.

Data exclusions	No data were excluded from the analyses.
Reproducibility	The complementary molecular, manipulative and census-based experiments provide confidence that the patterns observed in the data are broadly applicable. The long-term field investigation on seedling dynamics, which was undertaken at unprecedented temporal and spatial scales (17,824 individuals over ten years), provided reliability and reproducibility on the phenomena we observed. The manipulative seedling experiments and molecular analysis further provided mechanistic explanations.
Randomization	For both experiments, the cores were randomly distributed within a 3 × 2 m area in the centre of each site, with a random distance of 30 to 50 cm between neighboring cores.<br>We randomly selected and transplanted the seedlings into the mesh cores.<br>At the harvest, we randomly collected 10 fine root fragments of 1 cm length from each seedling for laboratory analysis.
Blinding	Blinding is not relevant to our study. We collected field data by recording the status, species identity, and height of all woody seedlings, and did not exclude any data for analysis.

Did the study involve field work? ☒ Yes ☐ No

Field work, collection and transport

Field conditions	The field site consists of 4200 ha of subtropical evergreen broad-leaved forest, which is located on the Tropic of Cancer and has a subtropical moist monsoon climate. Mean annual temperature is 19.6 °C and mean monthly temperatures range from 10.6 °C in January to 28.4 °C in July. Annual precipitation is about 1744 mm, occurring mainly between April and September (79% of annual rainfall), and a pronounced dry season lasts from October to March.
Location	The Heishiding Nature Reserve in Guangdong Province of south China (111°53'E, 23°27'N, 150-927 m a.s.l.)
Access and import/export	We have full access to the field site and the field facilities (shade houses and labs), and do not need to get permission to collect data. The field site is officially supported by the Ministry of Education of China, and managed by the School of Life Sciences, Sun Yat-sen University. No samples were exported overseas.
Disturbance	We inserted 168 in-growth cores in the soil at the study area, which were assembled from 16 cm diameter × 30 cm deep PVC piping. At the end of the experiments, all the PVC cores were removed from the soil and recycled. The holes were recovered with field soil.

Reporting for specific materials, systems and methods

We require information from authors about some types of materials, experimental systems and methods used in many studies. Here, indicate whether each material, system or method listed is relevant to your study. If you are not sure if a list item applies to your research, read the appropriate section before selecting a response.

Materials & experimental systems

n/a	Involved in the study
☒	☐ Antibodies
☒	☐ Eukaryotic cell lines
☒	☐ Palaeontology
☒	☐ Animals and other organisms
☒	☐ Human research participants
☒	☐ Clinical data

Methods

n/a	Involved in the study
☒	☐ ChIP-seq
☒	☐ Flow cytometry
☒	☐ MRI-based neuroimaging
